# Long-term results of breast conservation and immediate volume replacement with myocutaneous latissimus dorsi flap

**DOI:** 10.1186/1477-7819-9-159

**Published:** 2011-12-05

**Authors:** Fernando Hernanz, Sonia Sánchez, María Pérez Cerdeira, Carlos Redondo Figuero

**Affiliations:** 1 Breast Unit. Hospital, University of Cantabria, "Marqués de Valdecilla" (Avda Valdecilla s/n), Santander, (39008), Cantabria, Spain; 2 Department of Radiology. University of Cantabria, "Marqués de Valdecilla" (Avda Valdecilla s/n), Santander, (39008), Cantabria, Spain; 3 Department of Surgery. University of Cantabria, "Marqués de Valdecilla" (Avda Valdecilla s/n), Santander, (39008), Cantabria, Spain; 4 Faculty of Medicine, University of Cantabria, Avda Cardenal Herrera Oria s/n, Santander,(39011), Cantabria, Spain

**Keywords:** Oncoplastic breast surgery, long-term outcomes, cosmetic outcome and latissimus dorsi flap

## Abstract

**Background:**

Published long-term outcomes of oncoplastic breast-conserving surgery are scarce and, specifically, aesthetic outcomes assessed with an objective method have not previously been published.

**Methods:**

A cohort of 41 patients treated with a quadrantectomny and immediate reconstruction using a myocutaneous latissimus dorsi flap were analyzed and their aesthetic outcomes were evaluated objectively by BCCT.core software.

**Results:**

At the end of a 58-month follow-up from the date of initial diagnosis, one patient (2.4%) developed an ipsilateral recurrence, six patients developed distant metastases and three patients died (7.3%) without ipsilateral recurrence, one of them presenting hepatic metastases at the time of the initial diagnosis. We were able to evaluate aesthetic results in 23 patients, 3 assessed as excellent, 12 good and 8 fair.

**Conclusion:**

This oncoplastic volume replacement technique obtained a good local control and satisfactory and stable aesthetic results which have maintained unchanged after a long period of time.

## Background

Oncoplastic breast-conserving surgery (OBS) has become a gold standard in the surgical treatment of early breast cancer increasing the rate of breast conserving treatment (BCT), avoiding mastectomies and cosmetic sequelaes, and improving patients' quality of life and self-esteem.

Aesthetic outcome and degree of patient satisfaction is related to the percentage of breast tissue excised so when the preoperative estimation of this volume exceeds 20% of the total breast volume a reconstructive technique is required to obtain a good cosmetic outcome [1-3]. This option allows the achievement of the OBS goal: complete removal of the lesion, clear margins, the larger the better, good to excellent cosmetic result and operating once so as to perform the definitive procedure avoiding a complicated breast reconstruction postmastectomy process [4].

We published our experience using an OBS volume replacement technique (OVR), a real quadrantectomy and immediate reconstruction with myocutaneous latissimus dorsi flap (LDF) in one-stage procedure, analyzing the early results. The technique showed its utility allowing extensive resection, extending BCT to tumors with a mild response or no response after neoadjuvant chemotherapy, achieving good cosmetic outcomes and, in short, it could be particularly useful when radiotherapy indication, in the event of a mastectomy, were present before surgery or likely after surgery based on pathological features, such as more than three involved lymph nodes or bad prognostic factors [5-7]. Our experience with this technique obtained good early cosmetic results showing that symmetry and the quality of the reconstructed breast scar were the most influential factors in determining the cosmetic result [6].

The lack of randomized trial data makes comparison of OBS techniques difficult; most studies involve small cohorts of patients assessed for outcome in various ways and as far as aesthetic results are concerned, these are heterogeneous and difficult to evaluate in a standardized way [8]. A computer system (BCCT.core) has been developed to objectively and automatically evaluate the aesthetic result of BCT and it is has been proposed as a gold standard method for assessment of breast cosmesis in clinical trials as standard [9].

Some factors can deteriorate the early aesthetic outcome: the effect of radiotherapy reducing breast size and increasing the fibrotic, changes in body weight, and aging and gradual breast ptosis, which would worsen the cosmetic result increasing breast asymmetry. This has been experienced by Gendy, who observed an increment of the cosmetic failure rate from 10 to 18%, in a period of 43 months, in patients treated with a partial mastectomy with latissimus dorsi miniflap reconstruction [10].

This article aims to evaluate the long-term results, oncologic and aesthetic, of a cohort of patients treated with an OVR and observe the effect of time on the cosmetic results.

## Methods

We reviewed the records and radiological images of 41 patients treated with partial mastectomy (quadrantectomy or sector mastectomy) and immediate breast reconstruction by LDF (one-stage procedure) from November 2002 to May 2010; the patients' characteristics are described in table 1.

**Table 1:** Characteristics of 41 patients.

Characteristic	Value	IC-95%
**Age (years)**		
Median (SD)	**44 (7.5)**	**41.7 to 46.3**
Range	**22-58**	

**Breast volume^a^ (cc)**		
Median (SD)	**705.4 (294.0)**	**605.1 to 805.7**
Patient with breast volume n, (%)		
< 500	**8 (24.2)**	**12.8 to 41.0**
500-1000	**22 (66.7))**	**49.6 to 80.2**
< 1000	**3 (9.1)**	**3.1 to 23.6**
NC	**8**	

Size of tumor* (mm)		
Median (SD)	**22 (11.25)**	**18.4 to 25.6**
Range	**0-45**	

**Estimated volume of resection ° (cc)**		
Median (SD)	**47 (36.33)**	**35.3 to 58.7**

**Percentage of total breast volume**		
Median (SD)	**8.5 (7.66)**	**5.8 to 11.3**
Patients n, (percentage)		
0-10%	**21 (70)**	**52.1 to 83.3**
10-20%	**5 (16.7)**	**7.3 to 33.6**
> 20%	**4 (13.3)**	**5.3 to 29.7**
NC	**11**	

**Distribution of tumor through the breast (no. patients)**		
Upper outer quadrant	**19**	
Inferior inner quadrant	**1**	
Inferior outer quadrant	**3**	
Intersection upper quadrants	**9**	
Intersection inferior quadrants	**2**	
Intersection outer quadrants	**4**	
Intersection inner quadrants	**3**	

**Multifocal**		
Yes	**8 (19.5)**	**10.2 to 34.0**
No	**33 (80.5)**	**66.0 to 89.8**

**Histologic subtype of invasive carcinoma (no. of patients)**		
Ductal	**27**	
Lobular	**10**	
Solid	**1**	
Apocrine	**1**	
Phyllodes	**1**	
Mixed	**1**	

^a^Volume of the breast was calculated using the formula 1/3 pi H R_cc_R_ol_, (H) height and (R_ol_) radius measurements were taken from oblique mammogram projection and R_cc _radius from craneocaudal one.

* Tumor size was defined as the largest dimension recorded on mammogram or magnetic resonance imaging (MRI); in 19 patients who were treated preoperatively with chemotherapy this measurement was taken from the MRI or mammogram once the treatment was finished or interrupted.

°Estimated volume of resection was determined using the formula 4/3 pi (tumor size/2 + 1)^3^.

SD: standard deviation, CI: Confidence interval, NC: not confirmed

We calculated the estimated breast volume (V_br_) of 33 patients based on mammogram measurements using a modification of the Katariya method [11] assuming the elliptical cone projection with the formula:

$$V_{\mathrm{br}}=1∕3\;\mathrm{pi}\;R_{\mathrm{cc}}\;R_{\mathrm{ol}}\;H_{\mathrm{ol}}$$

where (H_ol_) height and (R_ol_) radius measurements were taken from oblique mammogram projection and R_cc _radius from craneocaudal projection.

Tumor size was defined as the largest dimension recorded on mammogram or magnetic resonance imaging (MRI); in those patients who were treated preoperatively with chemotherapy this measurement was taken from the MRI or mammogram once the treatment was finished or interrupted. The estimated volume of resection (V_ers_) was determined assuming that theoretical resection is a sphere containing the tumor in its core surrounded by 1 cm of healthy breast tissue (margin):

$$V_{\mathrm{ers}}=4∕3\;\mathrm{pi}\;R^{3}$$

where R is equal tumor size/2 + 1 cm.

All the patients underwent oncologic and reconstructive procedures, carried out by a complete or comprehensive oncoplastic breast surgeon (HF). In one patient a reductive contralateral mammaplasty was performed concomitantly to obtain symmetry. Table 2 shows pathologic features; surgical specimen volume was calculated from specimen weight using the density of 0.958 g/cm^3^. Positive margins were defined as having tumor cells right at the cut edge of the specimen. Close margins were defined as having tumor cells between the cut edge of the specimen and the boundary defined as negative (>2 mm). All the patients in this series received standard adjuvant radiotherapy, 50 Gy spread over four weeks, adjuvant systemic chemotherapy and endocrine therapy, which was indicated according to our standard protocol based on clinical and pathological findings.

**Table 2:** Characteristics of the surgical specimen (breast tissue and axillary lymph nodes)

Characteristic	Value	IC 95%
**Weight, g**		
Median (SD)	**174.8 (70.9)**	**152.5 to 197.0**
Range	**42-381**	

**Volume^a^ (cc)**		
Median (SD)	**167.4 (67.9)**	**146.1 to 188.7**
Range	**40-364**	

**Percentage of total breast volume**		
Median (SD)	**24.7 (8.7)**	**21.6 to 27.8**
Patients (IC)		
0-10	**0**	0.0 **to 11.0**
10-20%	**12 (38.7)**	**23.7 to 56.2**
> 20%	**19 (61.3)**	**43.8 t0 76.3**
NC	**10**	

**Margin, patients (percentage)**		
Involved, tumors cells	**0**	**17.6 to 44.5**
Close,< 2 mm	**12 (29.3)**	**55.5 to 82.4**
Negative, no tumors cells	**29 (70.7)**	

**Minimal width of margin, mm**		
Median (SD)	**5.2 (4.4)**	**3.8 to 6.6**

**Axillary lymph nodes**		
Number of patients with positive lymph nodes, n (percentage)	**20 (52.6)**	**37.3 to 67.5**
Number of patients with >3 positive lymph nodes n (percentage)	**9 (23.7)**	**13.0 to 39.2**

**Positive hormone receptors, n (percentage)**		
Estrogenic	19	**19.6 to 47.0**
Progesterone	22	
Herb-2	11	
**Triple-negative, n (percentage)**	**13 (31.7)**	

NC: not confirmed, CI: Confidence interval

Minimum follow-up was 13 months with an average of 58 from the date of initial pathological diagnosis until the date of the last study follow-up. All patients were followed-up clinically every 6 months for the first 3 years and then every year thereafter. Complete clinical examination, bilateral mammograms, chest X-rays, abdomino-pelvic ultrasonography and tumor markers were performed in each revision. Bone scans were indicated only in the event of suspicious blood tests or clinical symptoms.

The end point analyzed was the status (alive or dead, cause of death, free of disease or with recurrence, type of recurrence and survival) at the date of the last follow-up study.

### Assessment of cosmetic outcome

Cosmetic outcome was evaluated by BCCT.core software. Standardized digital front photographs were taken of naked patients standing up straight with their arms down beside the body after an average of 55 months from the date of the end of radiotherapy. BCCT.core (breast cancer conservative treatment cosmetic results) software was developed by The University of Porto to evaluate cosmetic results of BCT in an objective standardized semiautomatic way, and divides the result into four categories (excellent, good, fair and bad) [9].

Patients were also asked about changes in the sensitivity of the nipple of the affected breast, chronic back pain or functional limitations of the shoulder during their domestic activities.

### Statistical analysis

We calculated confidence intervals (CI) of proportion by means of the Wilson method [12]. We study the correlation between V_ers _and the actual volume excised_. _Values of p < 0.05 were considered significant.

## Results

**Figure 1:**
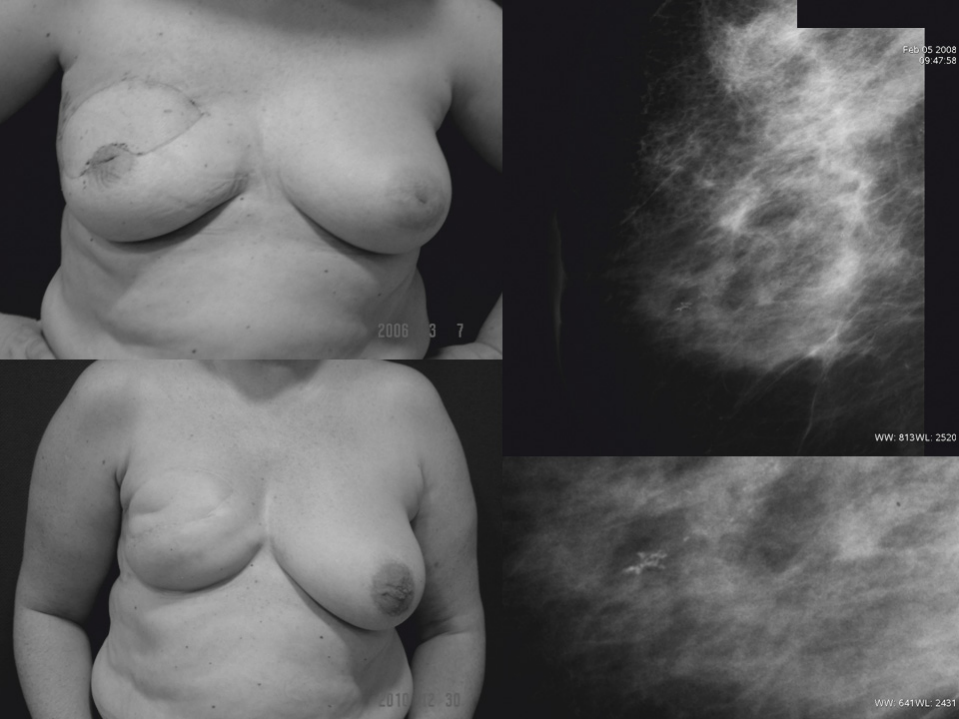
**Appearance of the patient who suffered from an ipsilateral breast cancer recurrence, she underwent a skin-sparing mastectomy and immediate breast reconstruction with an adjustable breast implant**. She did not want undergo a reduction mammaplasty for simmetrization. Mammography showed a small group of microcalcifications; the core biopsy confirmed an in situ ductal carcinoma.

Median follow-up was 58 months (13-95) and only two patients were lost at 16 and 64 months into the follow-up. One patient developed an ipsilateral breast cancer recurrence (2.4%), whose histological type was in situ ductal carcinoma, diagnosed 32 months after the date of the histological diagnosis. There were several factors that could have influenced this oncologic outcome, such as her young age (she was 44 years old), premenopausal status, the specific characteristics of her tumor: negative estrogen and progesterone receptors, positive Herb-2, and close margin caused by bifocal residual disease after neoadjuvant chemotherapy with a microfoci separated from the main tumor. She underwent a skin-sparing mastectomy and immediate breast reconstruction with an adjustable breast implant and she is alive and free of disease after 63 months' follow-up. (Figure 1)

Six patients developed distant metastases (14.63%), at an average time of 30 months (19-46) after the date of histological diagnosis, the metastases sites were: bone, lung, liver and lymph nodes, and three patients died (7.3%) due to the tumor without ipsilateral recurrence, one of them presenting hepatic metastases at the time of treatment. One patient developed a gastric cancer and another was diagnosed with contralateral breast cancer.

We were able to interview and evaluate 23 patients cosmetically, the reasons for this being: three patients died, one had an ipsilateral recurrence and was mastectomized, three had a distant recurrence and were in a bad state, one was diagnosed and treated for contralateral breast cancer and one had a surgical procedure on the affected breast, while the rest refused to participate or were impossible to contact. Table 3 shows cosmetic evaluation results and the changes from the previous evaluation [6,7]. In 19 patients we observed the evolution of the cosmetic outcomes, which had previously been assessed subjectively by a mixed panel in 2005 or 2007; four of them (21.05%) have changed their assessment deteriorating from good to fair.

**Table 3:** Aesthetic results.

Method of Evaluation	EXCELLENT	GOOD	FAIR	BAD	total
Panel, 2005	2 (6.8)	15 (51.7)	11 (37.9)	1 (3.4)	29

BCCT.core, 2010	3 (13.0)	12 (52.2)	8 (34.8)	0	23

n (percentage)

No patients expressed functional limitation in activities of daily life and, it is worth commenting that three women regularly go swimming, two or three times a week. One expressed a decrease in nipple sensation and another had chronic back pain.

## Discussion

Breast surgery and, in particular, OBS should be an individual process for each patient; the appropriate surgical technique requires that the surgeon should choose between the different options, weighing up the pros and cons of each one. Multiple reconstructive techniques are available for partial breast reconstruction, the dominant criterion used for selection is the location of the tumor but factors such as breast size, degree of ptosis and density of the breast tissue should be taken into account [13]. The patient's condition and preferences, characteristics of the tumor [14], for example, associated component in situ, lobular histologic type, and the level of the surgeon's expertise in OBS training also determine the choice [15,16].

Reviewing articles about OBS, more specifically those published in the last years, it can be observed that displacement techniques are more frequently used than volume replacement ones [17,18], one explanation might be that some surgeons are reluctant to spend a distant flap, such as LDF, for a partial breast reconstruction, concerned that this could potentially compromise a reconstructive option in situations in which completion mastectomy is required. They prefer other options like skin sparing mastectomy with immediate reconstruction in those patients with small or medium breast size where tumor excision causes large defects and reshaping is not possible.

However, we consider that OVR are very useful when the indication of radiotherapy postmastectomy is sure or quite likely; in this situation, this option offers more advantages than the skin sparing mastectomy with immediate reconstruction. The procedure only requires one surgical intervention, which takes less time, does not need surgery on the contralateral breast and the patients conserve their own nipples without changes in sensation. In our series almost half of the patients had had radiotherapy indication if a mastectomy was carried out based on the initial size of the tumor or on the lymph node status.

Although new OVR techniques based on artery perforator flaps [19] as thoracodorsal flaps [20] have gained acceptance in the reconstruction options because they could replace the classic LDF not sacrificing this muscle and avoiding common donor site morbidity and postoperative seroma, the LDF remains as preferential option in some clinical situations as central or medial defects in patients with small or moderate size breast (bra cup size A,B,C) [21,22].

**Figure 2:**
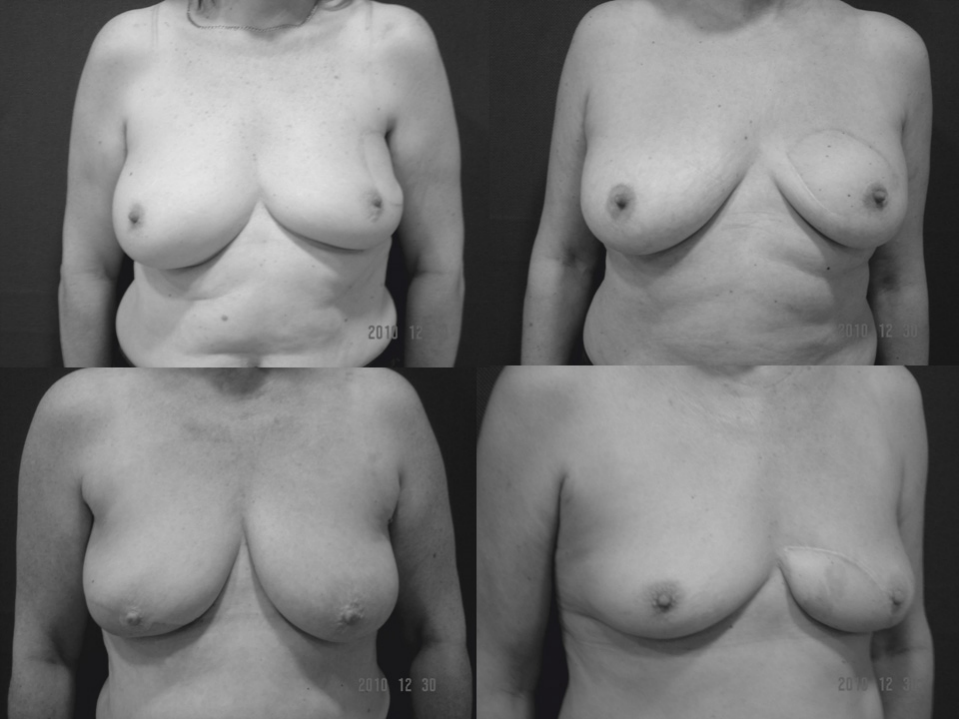
**Long-term aesthetic results of OVR used in the four quadrants of the breast**.

Outcomes of OBS are scarce and much more if we are dealing with OVR. In 2007 and 2008 Asgeirsson KS and Rainsbury [17,18] published a review of seven series with a total of 189 patients with a follow-up of 24 to 53 months showing a low local recurrence rate (0 to 5%) and cosmetic failure rate (0 to 18%); our work obtained similar rates which fall into the range of this series but adds some more information about one OVR, the partial mastectomy with immediate LDF reconstruction, the usefulness in all quadrants of the breast achieving acceptable and stable aesthetic outcomes over a long period of time (Figure 2).

Our series can be characterized with some data: patients below the average age of breast cancer (44 years) with medium size breasts (705 cc average of total breast volume) suffered from tumors with 22 mm of average size (22 after neoadjuvant chemotherapy) located in all quadrants of the breast, multifocal in 20% cases, with high rate of axillary lymph node affectation (46.3%) and triple-negative receptors (31.7%). The OVR used achieved appropriate margins (no patients had involved ones) and a good local control (rate of ipsilaterall recurrence of 2.4%) but significant discordance between the estimated volume of resection (6.6% of the breast volume) and the actual volume excised (24.7%) was observed (correlation coefficient, r = 0.0195, p = 0.321). The explanation for this is the surgical specimen, a quadrantectomy or sector partial mastectomy, which does not fit a sphere containing the tumor in the core so some healthy breast tissue is unnecessarily taken out. Despite the high breast volume excised, with an average of 167 cc, which is similar to other published data (23, 24), the cosmetic results were not negatively affected.

Cosmetic outcome in OBS evaluated with an objective method have not been published previously. Aesthetic outcomes evaluated by BCCT.core are worse than when they are evaluated by a panel [25], but this is an objective and easily reproducible method which offers us the opportunity to standardize the evaluation allowing a fair comparison between different studies.

Cosmetically satisfactory results were achieved in 65% of cases after a long follow-up period 54 months (range from 5 to 92), similar results have been published by other authors such as Naguib SF [26], who published 69% in a series of 29 patients, in which LDF was used in 21 cases, and 16 kept their NAP after a follow-up ranging from 3 to 36 months and Tomita K [27], who achieved 75% good cosmetic results in 44 patients evaluated by a mixed panel one year after the end of treatment.

**Figure 3:**
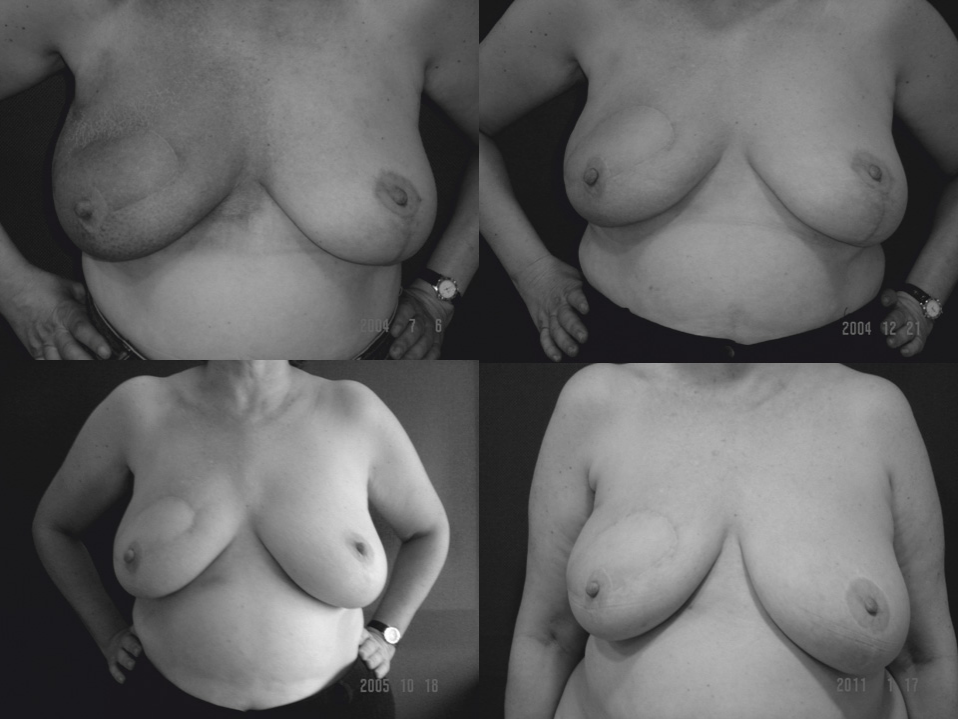
**Appearance of the patient at different times, while the radiotherapy was delivered and, three months, two years and five years after the end of radiotherapy**.

In 19 patients we observed the evolution of the cosmetic outcomes because they were assessed subjectively by a mixed panel in 2005 or 2007. Only four patients have changed their assessment with deterioration from good to fair (Figure 3). The main reason that could explain this worsening is the increment of the asymmetry, which was together with the scar of the reconstructed breast the main factor determining the aesthetic result [6,28]. Like other breast surgeries (reconstruction, reduction and augmentation), changes in the weight status worsen the aesthetic results as we could see in three of the four patients whose aesthetic results worsened, two having put on weight and the other, who suffered from a second neoplasia, an advanced gastric carcinoma, losing weight dramatically.

Finally, we observed that the technique does not cause a late morbidity and the patients have a complete adjustment and do not have limitations in everyday life.

## Conclusion

This OVR obtained a good local control and satisfactory and stable aesthetic results which have maintained unchanged after a long period of time. This option should be considered when the postmastectomy radiotherapy indication is present or likely

## List of abbreviations used

OBS: Oncoplastic breast surgery; BCT: Breast conserving treatment; OVR: OBS volume replacement technique; LDF: Myocutaneous latissimus dorsi flap; MRI: Magnetic resonance imaging; CI: Confidence intervals.

## Competing interests

The authors declare that they have no competing interests.

## Authors' contributions

HF, general surgeon who carried out the surgical procedures and principal investigator, participated in design and coordination of the study

SS, radiologist who participated in data collecting and reviewed radiological images

P-CM, general surgeon who participated in data collecting and surgical procedures

R-FC, physician and statistic who conducted statistical analysis

All authors read and approved the final manuscript
